# Artificial Intelligence in Aquatic Biodiversity Research: A PRISMA-Based Systematic Review

**DOI:** 10.3390/biology14050520

**Published:** 2025-05-08

**Authors:** Tymoteusz Miller, Grzegorz Michoński, Irmina Durlik, Polina Kozlovska, Paweł Biczak

**Affiliations:** 1 Institute of Marine and Environmental Sciences, University of Szczecin, 71-415 Szczecin, Poland; grzegorz.michonski@usz.edu.pl; 2 Polish Society of Bioinformatics and Data Science, Biodata, 71-214 Szczecin, Poland; i.durlik@pm.szczecin.pl (I.D.); pawel@internationalsafetyadvisors.com (P.B.); 3 Faculty of Navigation, Maritime University of Szczecin, 70-500 Szczecin, Poland; 4 Faculty of Economics, Finance and Management, University of Szczecin, 71-412 Szczecin, Poland; 231805@stud.usz.edu.pl

**Keywords:** artificial intelligence, aquatic biodiversity, machine learning, deep learning, species identification, habitat modeling, conservation, ecological monitoring, freshwater ecosystems

## Abstract

Freshwater ecosystems are increasingly threatened by climate change and anthropogenic activities, necessitating innovative and scalable monitoring solutions. Artificial intelligence (AI) has emerged as a transformative tool in aquatic biodiversity research, enabling automated species identification, predictive habitat modeling, and conservation planning. This systematic review follows the PRISMA framework to analyze AI applications in freshwater biodiversity studies. Using a structured literature search across Scopus, Web of Science, and Google Scholar, we identified 312 relevant studies published between 2010 and 2024. This review categorizes AI applications into species identification, habitat assessment, ecological risk evaluation, and conservation strategies. A risk of bias assessment was conducted using QUADAS-2 and RoB 2 frameworks, highlighting methodological challenges, such as measurement bias and inconsistencies in the model validation. The citation trends demonstrate exponential growth in AI-driven biodiversity research, with leading contributions from China, the United States, and India. Despite the growing use of AI in this field, this review also reveals several persistent challenges, including limited data availability, regional imbalances, and concerns related to model generalizability and transparency. Our findings underscore AI’s potential in revolutionizing biodiversity monitoring but also emphasize the need for standardized methodologies, improved data integration, and interdisciplinary collaboration to enhance ecological insights and conservation efforts.

## Simple Summary

This review explores the emerging role of artificial intelligence in supporting aquatic biodiversity research. We present a structured analysis of how AI techniques—such as machine learning, deep learning, and transformers—are applied to key ecological tasks, including species identification, habitat modeling, ecological risk assessment, remote sensing analysis, and conservation planning. For each domain, we link biological questions to computational problems and discuss the suitability and limitations of specific AI algorithms. We also highlight real-world case studies and provide a methodological classification of AI approaches based on the data type and modeling goal. This work offers both ecologists and data scientists a comprehensive perspective on how AI can advance biodiversity monitoring and support conservation strategies in freshwater ecosystems.

## 1. Introduction

Freshwater ecosystems, including rivers, lakes, wetlands, and groundwater systems, are among the most biologically diverse environments on Earth. Despite covering only about 2.5% of the planet’s total water resources, they support nearly 10% of all known species, including a vast array of fish [1], amphibians, invertebrates, and aquatic plants. These ecosystems play a crucial role in maintaining ecological balance, providing essential services, such as water purification, flood regulation, and carbon sequestration [2].

However, freshwater biodiversity is under severe threat due to climate change, habitat destruction, pollution [3], invasive species, and overexploitation. According to recent reports, freshwater species populations have declined by nearly 84% since 1970, making freshwater ecosystems among the most endangered on the planet. Monitoring and conserving biodiversity in these habitats is therefore a global priority, requiring innovative and scalable approaches to assess ecosystem health and implement conservation strategies [4].

Artificial intelligence (AI) has emerged as a powerful tool in biodiversity research, revolutionizing traditional monitoring methods by enabling automated species identification [5,6], habitat mapping, and ecological risk assessment [7]. Machine learning (ML) and deep learning (DL) techniques, particularly computer vision, bioacoustics, natural language processing (NLP), and remote sensing AI models, have significantly enhanced the accuracy and efficiency of biodiversity studies [8].

Key AI applications in aquatic biodiversity research include:

1. Species identification using convolutional neural networks (CNNs) for image recognition.
2. Bioacoustic analysis for detecting species presence through underwater sound recordings.
3. Predictive habitat modeling for assessing ecosystem changes due to climate variability.
4. Ecological risk assessment to evaluate pollution impact and detect environmental threats [9].
5. Remote sensing AI for large-scale biodiversity monitoring using satellite and drone imagery.

These AI-driven approaches offer unprecedented capabilities for processing vast amounts of ecological data, enabling researchers to develop more accurate models for biodiversity conservation, policymaking, and ecosystem management [10]. However, despite its potential, AI applications in freshwater biodiversity research still face challenges related to data availability, model generalization, ethical concerns, and the need for standardized methodologies.

The primary objective of this review is to provide a comprehensive synthesis of the current state of AI applications in freshwater biodiversity research, following the Preferred Reporting Items for Systematic Reviews and Meta-Analyses (PRISMA) framework. Specifically, this study aims to:

1. Systematically analyze AI-based methodologies applied to freshwater biodiversity monitoring and conservation.
2. Categorize AI applications into key areas, such as species identification, habitat modeling, ecological risk assessment, and conservation strategies.
3. Evaluate methodological strengths and limitations, including risk of bias, data quality issues [11], and validation challenges.
4. Identify citation trends and geographical contributions in AI-driven biodiversity research.
5. Highlight knowledge gaps and propose future research directions to improve AI’s role in freshwater biodiversity conservation.

This systematic review is intended to serve as a reference for researchers, ecologists, and policymakers, facilitating the development of more robust, scalable, and effective AI-based conservation strategies to protect and restore freshwater biodiversity.

## 2. Methodology (PRISMA Framework)

### 2.1. Literature Search Strategy

This systematic review was conducted following the Preferred Reporting Items for Systematic Reviews and Meta-Analyses (PRISMA) guidelines. The literature search was performed across Scopus, Web of Science, and Google Scholar databases, ensuring comprehensive coverage of AI applications in aquatic biodiversity research. The following Boolean search string was utilized to retrieve relevant publications:

(TITLE-ABS-KEY(“artificial intelligence” OR “machine learning” OR “deep learning” OR “computer vision” OR “natural language processing” OR “AI”) AND TITLE-ABS-KEY (“aquatic biodiversity” OR “freshwater biodiversity” OR “water quality” OR “species identification” OR “habitat modeling” OR “ecological monitoring”) AND TITLE-ABS-KEY (“conservation” OR “risk assessment” OR “environmental impact” OR “ecological modeling”)) AND PUBYEAR > 2010 AND PUBYEAR < 2025 (https://www.scopus.com/results/results.uri?sort=plf-f&src=s&sid=ea56e0dd621c4d30fcb500e9e1857c03&sot=a&sdt=a&sl=461&s=%28TITLE-ABS-KEY%28%22artificial+intelligence%22+OR+%22machine+learning%22+OR+%22deep+learning%22+OR+%22computer+vision%22+OR+%22natural+language+processing%22+OR+%22AI%22%29+AND+TITLE-ABS-KEY%28%22aquatic+biodiversity%22+OR+%22freshwater+biodiversity%22+OR+%22water+quality%22+OR+%22species+identification%22+OR+%22habitat+modeling%22+OR+%22ecological+monitoring%22%29+AND+TITLE-ABS-KEY%28%22conservation%22+OR+%22risk+assessment%22+OR+%22environmental+impact%22+OR+%22ecological+modeling%22%29%29AND+PUBYEAR+%26gt%3B+2010+AND+PUBYEAR+%26lt%3B+2025&origin=searchadvanced&editSaveSearch=&txGid=7494e69bf030c58a1e2071ffa8ca9026&sessionSearchId=ea56e0dd621c4d30fcb500e9e1857c03&limit=10, accessed on 6 February 2025).

This query was designed to ensure that only studies related to AI applications in freshwater biodiversity research were retrieved. The search was limited to studies published in peer-reviewed journals, conference proceedings, and institutional reports. No language restrictions were applied, but non-English papers were excluded if no translated version was available.

In constructing the search strategy, we employed Boolean logic using keywords such as “artificial intelligence”, “machine learning”, “deep learning”, “computer vision”, and “aquatic biodiversity”. While this strategy enabled us to capture a broad swath of the literature, we acknowledge the inherent risk of bias introduced by specific term selection.

For instance, the inclusion of “computer vision” may have skewed the results toward image-based applications (e.g., visual species recognition) while underrepresenting studies focused on environmental DNA (eDNA), natural language processing (e.g., ecological literature mining), or acoustic monitoring. To partially mitigate this, we performed a sensitivity analysis using additional terms (e.g., “eDNA”, “bioacoustics”, and “NLP”) and noted that while the overall thematic structure remained consistent, several relevant studies were indeed uncovered that had been missed by the initial query.

We recommend that future systematic reviews in this domain employ hybrid approaches that combine keyword-based searches with citation network exploration or machine-assisted discovery tools to reduce thematic bias and increase comprehensiveness.

### 2.2. Inclusion and Exclusion Criteria

The selection of studies was based on predefined inclusion and exclusion criteria to ensure relevance and quality. Studies were included if they applied AI techniques, such as machine learning, deep learning, natural language processing, or computer vision, to aquatic biodiversity research. Additionally, research needed to focus specifically on freshwater ecosystems, including rivers, lakes, and wetlands. Only studies providing empirical evidence or case studies that demonstrated AI applications in species identification, habitat modeling, or conservation strategies were considered for inclusion.

Several exclusion criteria were applied to refine the dataset. Studies that focused on marine biodiversity rather than freshwater ecosystems were excluded, as they fell outside the scope of this review. Papers that lacked a substantial AI methodology or did not clearly specify how AI was applied in their research were also omitted to maintain methodological rigor. Review articles that did not contribute original empirical findings were not considered, ensuring that this review remained focused on primary research. Furthermore, studies that did not validate AI model performance or lacked sufficient methodological transparency were excluded due to concerns regarding reliability and reproducibility. Lastly, non-English studies without an available translated version were not included to maintain consistency in the data analysis and interpretation. These exclusion criteria were applied systematically to ensure that only studies meeting high-quality standards contributed to the final synthesis.

### 2.3. Study Selection (PRISMA Flow Diagram)

**Figure 1:**
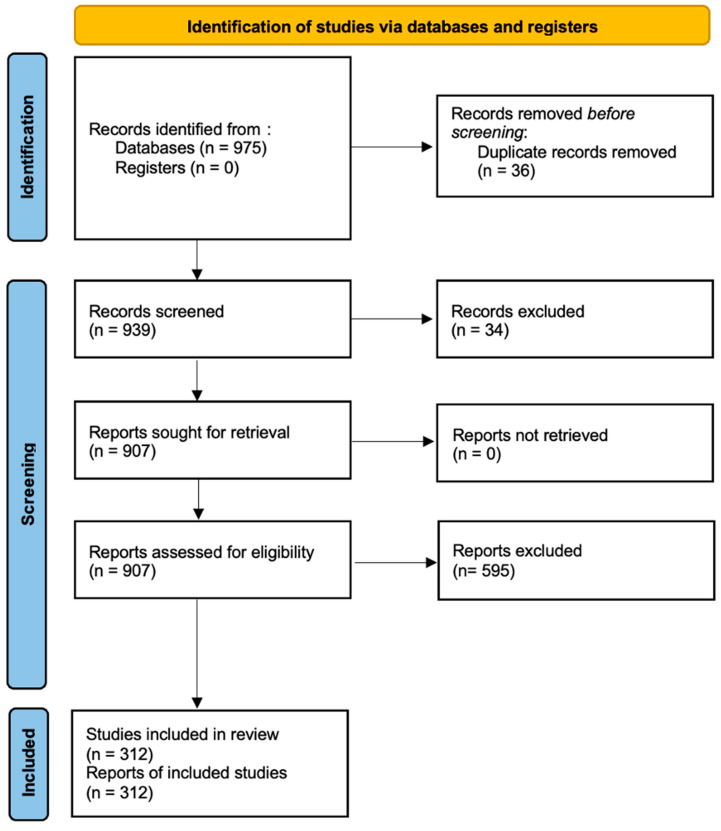
Prisma flow diagram.

The study selection process followed the PRISMA flow diagram, consisting of four main phases: identification, screening, eligibility assessment, and inclusion. The initial search yielded a total of 975 records. After removing duplicates and irrelevant studies based on title and abstract screening, 615 studies remained. A full-text review was conducted on these, leading to the final selection of 312 studies for inclusion in the systematic review (Figure 1).

**Figure 2:**
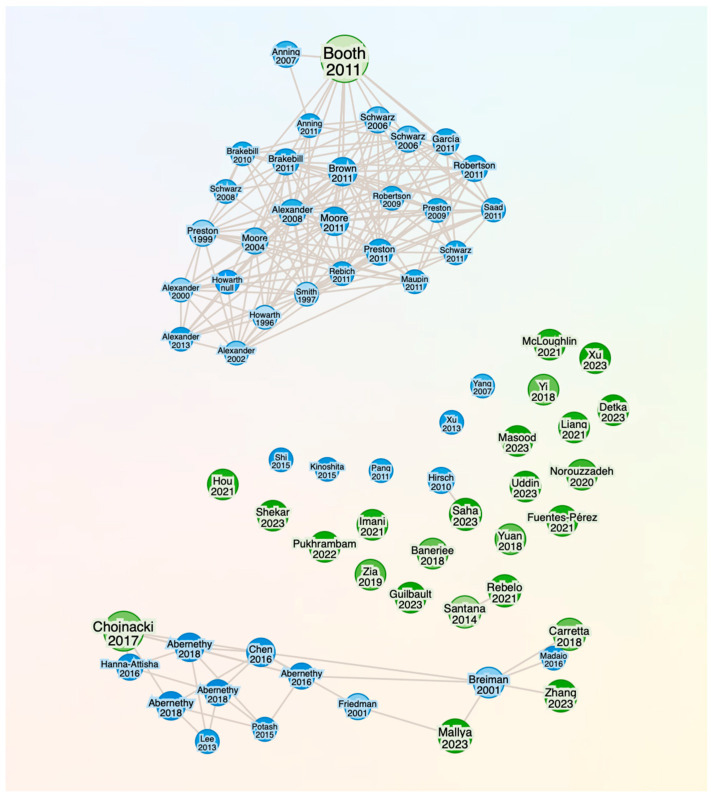
A citation network visualization centered on key studies in AI-driven aquatic biodiversity monitoring. The graph was generated using Research Rabbit and reveals thematic clusters, including foundational ecological modeling, machine learning theory and contemporary AI applications in species detection and environmental genomics.

To enrich the bibliometric analysis and ensure thematic breadth, we generated a citation network based on core studies identified during initial screening. Using Research Rabbit, we visualized how foundational and contemporary works are interlinked through citation patterns. As illustrated in Figure 2, the graph reveals multiple clusters, ranging from early ecological modeling frameworks to recent advances in AI-based monitoring, bioacoustics, and eDNA analytics.

This visual exploration confirmed the presence of cross-disciplinary bridges and highlighted several influential publications that were not highly ranked by a keyword search alone. Citation mapping thus served as a complementary tool to reduce thematic bias and identify structurally important but terminologically divergent contributions.

### 2.4. Assessment of Risk of Bias

To ensure the validity of findings, the assessment of the risk of bias was conducted using established frameworks. The QUADAS-2 tool was applied for diagnostic studies, and the RoB 2 tool was used for interventional research. Each study was evaluated across three key domains:

1. Selection bias: evaluated based on transparency in inclusion criteria and representativeness of study populations.
2. Information bias: assessed through data source reliability and robustness of AI methodologies.
3. Measurement bias: analyzed through the consistency and repeatability of AI model performance.

**Figure 3:**
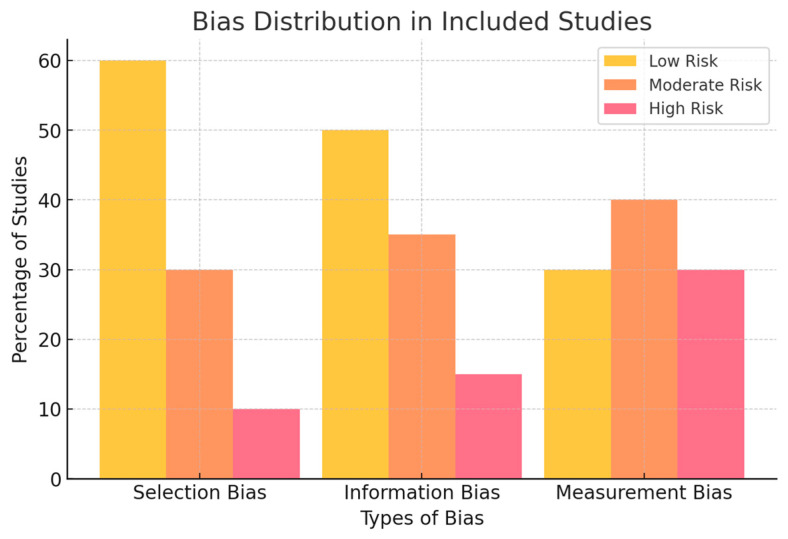
Summary of bias distribution.

The findings revealed that while selection and information bias were generally low across most studies, measurement bias was high in studies that lacked clear validation methodologies or relied on secondary data sources. A summary of bias distribution is illustrated in Figure 3.

Figure 3 illustrates the distribution of risk of bias across three domains: selection bias, information bias, and measurement bias. The majority of studies were rated as having low risk for selection bias (60%), indicating that inclusion criteria and population representativeness were generally well reported. Similarly, 50% of studies had low information bias, reflecting adequate data source reliability and clear methodological descriptions.

However, measurement bias emerged as a more prevalent issue, with only 30% of studies rated as low risk in this category. A notable proportion (40%) exhibited moderate measurement bias, and another 30% were rated as high risk. This trend highlights the frequent absence of external validation, inconsistent reporting of performance metrics (e.g., precision, recall), and a lack of reproducibility in AI model assessments. In some cases, performance evaluations relied solely on internal cross-validation or secondary datasets, further undermining reliability.

This distribution underscores a key limitation in current AI-driven aquatic biodiversity studies: while data collection and algorithm specification are increasingly standardized, the evaluation and reporting of model performance remain inconsistent. Future studies should adopt more transparent and rigorous validation protocols to mitigate measurement bias and improve the reproducibility of AI-based ecological findings.

A significant challenge in systematic reviews is the potential for publication bias, where studies reporting positive or significant findings are more likely to be published than those with null or negative results. This can skew the perceived effectiveness of AI applications in aquatic biodiversity research and limit the reliability of synthesized conclusions.

To mitigate this risk, additional searches were conducted beyond the traditional peer-reviewed literature. This included screening preprints from repositories, such as arXiv and bioRxiv, reviewing institutional reports and white papers from research organizations, and analyzing conference proceedings from AI and ecology-related conferences. These sources provided valuable insights into ongoing research that may not yet be formally published.

Despite these efforts, the potential for underreporting of negative AI outcomes remains a limitation of this review. Studies with inconclusive or unfavorable results may not be as readily accessible, potentially leading to an overestimation of AI’s effectiveness in biodiversity monitoring. To improve transparency and reduce reporting bias, future research should prioritize the use of comprehensive databases, encourage the publication of negative or neutral findings, and foster collaborations with research groups that promote open science initiatives. Standardized reporting guidelines for AI-driven ecological research could further support balanced representation of both successful and unsuccessful applications.

For QUADAS-2, we retained the four standard domains—patient selection, index test, reference standard, and flow and timing—and mapped them to AI-specific elements, as follows:

1. Patient selection was interpreted as the data sampling strategy, including whether datasets were balanced, representative, and clearly defined.
2. The index test corresponded to the AI model itself, including algorithm specification, tuning procedures, and availability of implementation details.
3. The reference standard referred to the ground truth quality—i.e., how labels were obtained and whether they were verified by domain experts.
4. Flow and timing were aligned with temporal consistency in data collection and model evaluation procedures.

We applied the following simplified thresholds during assessment (Table 1):

For the RoB 2 tool, the primary domains—randomization process, deviations from intended interventions, missing outcome data, measurement of outcome, and selection of the reported result—were similarly mapped. Given the non-randomized nature of most AI studies, we emphasized:

1. Bias due to missing data (e.g., unreported performance metrics);
2. Bias in outcome measurement (e.g., lack of precision/recall/F1-score reporting);
3. Selective reporting (e.g., absence of comparison with baseline models).

### 2.5. Data Extraction and Analysis

For each included study, a structured data extraction framework was used to ensure consistency. The extracted data included research objectives, methodologies, AI techniques, target species, and evaluation metrics. Studies were categorized thematically into the following:

1. AI advancements in species identification and biodiversity assessment.
2. Habitat modeling and ecological impact prediction.
3. Ethical and regulatory considerations of AI applications in conservation.

A mixed-methods approach combined quantitative metrics (e.g., citation frequency, model accuracy benchmarks) with qualitative insights from expert evaluations. This ensured a comprehensive synthesis of findings.

To enhance methodological clarity, we adopted a mixed-methods approach that combined both quantitative and qualitative analyses. This dual strategy enabled us to systematically assess trends in AI applications in aquatic biodiversity research while also interpreting the broader ecological and methodological contexts.

The quantitative component included descriptive statistics, such as publication counts over time, citation frequencies, geographic distribution of studies, model performance metrics (e.g., accuracy, precision, recall), and frequency of specific AI methods. These allowed us to establish macro-level patterns in the research landscape.

The qualitative component focused on thematic categorization of studies, evaluation of methodological rigor, ecological relevance of AI techniques, presence or absence of explainability tools (e.g., SHAP, LIMEs), and identification of ethical and practical challenges raised by the authors.

The two components were integrated during the synthesis phase (Section 3 and Section 4), where numerical findings were paired with contextual interpretation. For example, we combined citation data with model transparency ratings to assess which methods gained traction while maintaining ethical robustness. Similarly, regional trends were analyzed in light of biodiversity protection goals.

**Figure 4:**
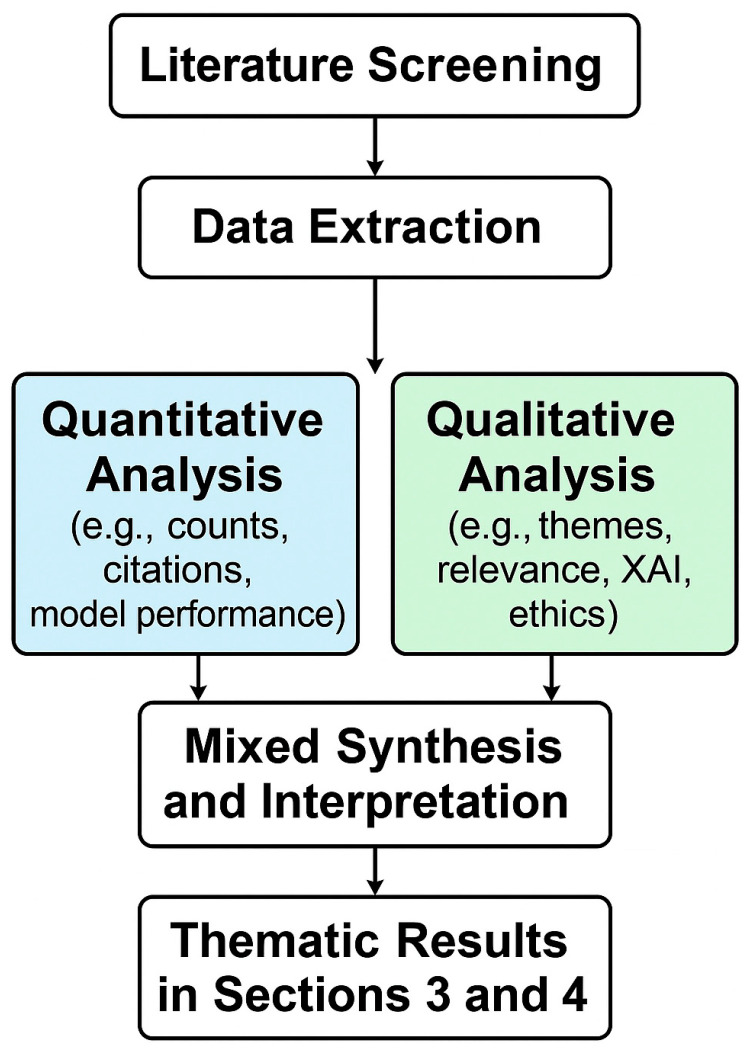
Integration flowchart.

To facilitate understanding, we created an integration flowchart (Figure 4) that maps the mixed-methods process, from data extraction to result synthesis. This visual representation helps clarify how quantitative and qualitative insights were blended to support evidence-based conclusions.

This approach strengthens the internal coherence of the review and supports a more holistic understanding of how AI tools are deployed in the service of biodiversity monitoring and conservation.

### 2.6. Citation Trends

**Figure 5:**
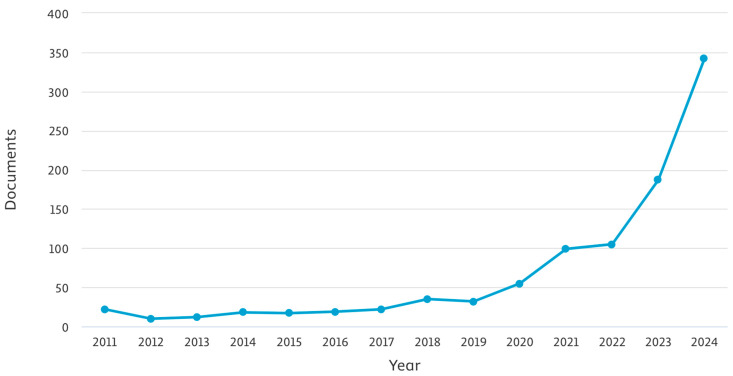
Publication trends.

The citation trends for artificial intelligence applications in aquatic biodiversity research exhibit a significant upward trajectory, particularly in the last five years. The number of research papers published per year has steadily increased, reflecting the growing integration of AI in ecological and environmental studies. In 2011, only 22 papers were published, with a modest rise in subsequent years. By 2015, the count reached 17, and by 2020, the annual number of publications had grown to 55. The rapid acceleration began in 2021 with 99 papers, followed by 105 in 2022, 187 in 2023, and a substantial surge to 342 publications in 2024. This pattern demonstrates a strong academic and research community focus on applying AI methodologies to address biodiversity challenges in freshwater ecosystems (Figure 5).

**Figure 6:**
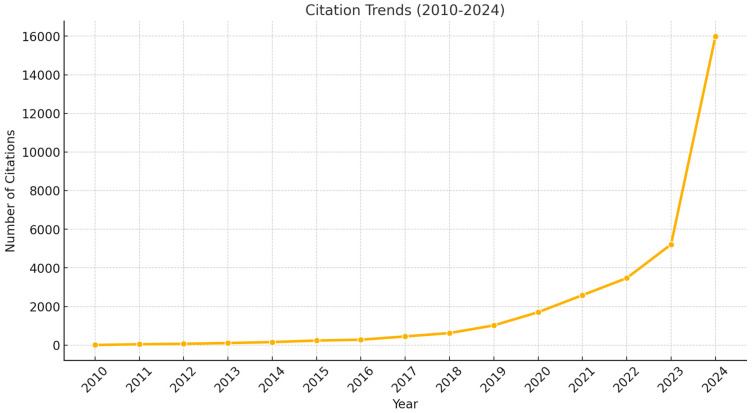
Citation trends.

The overall trend in citation numbers also shows a remarkable increase. From 7 citations in 2010, the count rose steadily to 45 in 2011, 69 in 2012, 109 in 2013, and 157 in 2014. A sharper rise was observed from 2015 onwards, with citations increasing to 237 in 2015, 280 in 2016, 450 in 2017, and 625 in 2018. The most significant growth occurred from 2019 to 2024, with citations reaching 1022 in 2019, 1705 in 2020, 2587 in 2021, 3472 in 2022, 5215 in 2023, and a peak of 15,980 in 2024, reflecting the increasing recognition and influence of AI-driven biodiversity research in the scientific community (Figure 6).

Regional contributions to the field reveal a dominant presence from technologically advanced and ecologically diverse nations. China leads with 209 publications, followed closely by the United States with 191, and India with 185. Other significant contributors include Australia (52), Germany (47), Canada (44), and the United Kingdom (43). Spain, South Korea, and France have also maintained active research output, each contributing between 36 and 42 papers. Notably, countries such as Saudi Arabia, Italy, and the Netherlands exhibit increasing engagement, suggesting a more global adoption of AI in biodiversity research.

**Figure 7:**
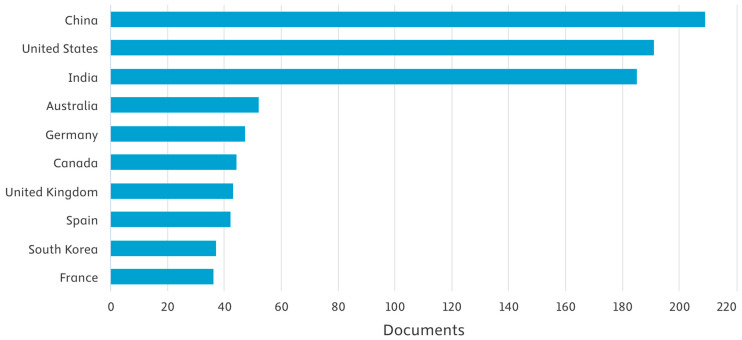
Regional contribution.

In Asia, Malaysia (24), Bangladesh (23), and Iran (22) show steady research activity. South Africa (23) and Brazil (17) represent the leading contributors from the Global South. European participation is further highlighted by Switzerland (21), Belgium (15), and the Czech Republic (14). Smaller but notable contributions come from countries like Taiwan, Turkey, Portugal, and Denmark, each producing over ten research papers. Interestingly, several emerging research hubs, including Chile, Ecuador, Hungary, and Iraq, have begun engaging in this interdisciplinary research domain, albeit at a lower volume (Figure 7).

Overall, the increasing publication rate and the broad geographic distribution of research efforts suggest a widespread recognition of AI’s potential in revolutionizing biodiversity monitoring and conservation efforts [12]. The growing impact of these studies underscores the necessity for interdisciplinary collaboration and methodological advancements to refine AI applications in aquatic biodiversity studies [13,14,15,16,17,18,19,20,21,22,23,24,25,26,27,28,29,30,31,32,33,34,35,36,37,38,39,40,41,42,43,44,45,46,47,48,49,50,51,52,53,54,55,56,57,58,59,60,61,62,63,64,65,66,67,68,69,70,71,72,73,74,75,76,77,78,79,80,81,82,83,84,85,86,87,88,89,90,91,92,93,94,95,96,97,98,99,100,101,102,103,104,105,106,107,108,109,110,111,112,113,114,115,116,117,118,119,120,121,122,123,124,125,126,127,128,129,130,131,132,133,134,135,136,137,138,139,140,141,142,143,144,145,146,147,148,149,150,151,152,153,154,155,156,157,158,159,160,161,162,163,164,165,166,167,168,169,170,171,172,173,174,175,176,177,178,179,180,181,182,183,184,185,186,187,188,189,190,191,192,193,194,195,196,197,198,199,200,201,202,203,204,205,206,207,208,209,210,211,212,213,214,215,216,217,218,219,220,221,222,223,224,225,226,227,228,229,230,231,232,233,234,235,236,237,238,239,240,241,242,243,244,245,246,247,248,249,250,251,252,253,254,255,256,257,258,259,260,261,262,263,264,265,266,267,268,269,270,271,272,273,274,275,276,277,278,279,280,281,282,283,284,285,286,287,288,289,290,291,292,293,294,295,296,297,298,299,300].

## 3. AI Applications in Aquatic Biodiversity Research

Artificial intelligence has emerged as a transformative force in biodiversity research, offering novel solutions for species identification [13], habitat assessment, ecological risk modeling [14], and conservation planning. By leveraging machine learning and deep learning techniques [15], researchers can analyze vast datasets, automate labor-intensive processes, and enhance the precision of biodiversity monitoring efforts [16]. This section explores the major applications of AI in aquatic biodiversity research, categorizing them into species identification [17], habitat and risk assessment, remote sensing applications, and conservation strategies (Table 2).

In this section, we examine key AI applications by structuring each analysis around the following elements: (i) the biological research question addressed, (ii) the computational modeling task derived from the problem (e.g., classification, regression, or clustering), (iii) the selection and suitability of AI and ML methods, and (iv) the limitations of the applied techniques. This framework allows for a deeper understanding of how AI methodologies align with specific ecological objectives, guiding future developments in aquatic biodiversity monitoring and conservation.

### 3.1. AI for Species Identification

Accurate species identification forms the cornerstone of aquatic biodiversity research, enabling ecological monitoring, population assessment, and conservation planning. The biological research question centers on whether species can be reliably and automatically identified based on observable data modalities, such as images, acoustic signals, or genomic sequences [18,19,20]. From a computational perspective, these challenges are typically formalized as supervised classification tasks, where the model must assign an input (e.g., an image, audio spectrogram, or DNA sequence) to a predefined taxonomic label.

Advances in machine learning and deep learning have substantially accelerated this process by minimizing reliance on manual taxonomy and mitigating observer bias [21,22,23,24,25]. Three principal modalities have emerged: image-based recognition using computer vision, bioacoustic monitoring with deep learning models, and genomic-based classification leveraging AI.

#### 3.1.1. Image-Based Species Recognition Using Computer Vision

Image-based species recognition addresses the biological question of whether aquatic organisms can be automatically identified from visual data, such as photographs or underwater videos. This challenge is typically approached as an image classification problem, where the goal is to map image data to species labels using machine learning models. Convolutional neural networks (CNNs) have become the dominant method in this domain [26,27]. Architectures such as ResNet, EfficientNet, and YOLO (You Only Look Once) are commonly used because of their ability to extract complex hierarchical features from visual inputs, achieving high classification accuracy even under challenging conditions [28,29,30].

YOLO models, in particular, enable real-time species detection and tracking in dynamic underwater environments [31,32,33]. CNNs are highly suitable due to their capacity for end-to-end feature learning, which eliminates the need for manual feature engineering. Transfer learning further enhances their utility by allowing models pre-trained on large datasets to be fine-tuned with limited aquatic data.

Nevertheless, these models require substantial volumes of labeled training data, which are often scarce in freshwater research [34,35,36]. Additionally, CNNs are typically black-box systems with limited interpretability, which may hinder biological validation. Their computational demands for training and fine-tuning also pose a barrier for smaller research groups.

#### 3.1.2. Bioacoustic Monitoring with Deep Learning

Bioacoustic monitoring explores the biological question of whether species presence and behavior can be detected and classified based on underwater sound recordings. In computational terms, this task is framed as time-series classification, where sequences of acoustic features—often derived from spectrograms—are mapped to species identities. Deep learning models, particularly recurrent neural networks (RNNs) such as Long Short-Term Memory (LSTM) and Gated Recurrent Units (GRUs), are widely used due to their capacity to capture temporal dependencies within sequential data [37,38,39,40]. These models are especially effective in distinguishing species-specific vocalizations and ecological events from environmental noise. By analyzing the broader temporal structure of acoustic signals, RNNs outperform traditional models that rely solely on static features [41,42,43].

However, the underwater acoustic environment presents numerous challenges, including variability caused by anthropogenic interference and overlapping calls between species [44,45,46]. Furthermore, RNNs are prone to overfitting when trained on small or imbalanced datasets, and their optimization requires substantial computational resources.

#### 3.1.3. DNA and eDNA-Based Species Identification

DNA and environmental DNA (eDNA) analysis addresses the biological question of whether aquatic species can be reliably detected and classified using genetic material extracted from environmental samples. The computational task involved is typically sequence classification, where nucleotide patterns are analyzed to infer species identity. Traditional machine learning algorithms, such as Support Vector Machines (SVMs) and Random Forests (RFs), have been successfully applied to classify species based on genetic markers obtained from DNA barcoding and eDNA analyses [47,48,49,50,51,52]. These models offer interpretability and robustness, particularly for structured, low-dimensional datasets. More recently, transformer-based architectures like DNABERT have emerged as powerful tools for modeling sequential and contextual properties of genetic sequences [53,54]. These models improve accuracy in distinguishing closely related or cryptic species by capturing long-range dependencies.

Despite their promise, transformer models require large, high-quality annotated datasets and are computationally intensive, which limits their adoption in aquatic biodiversity research [55]. Additionally, the lack of comprehensive reference libraries for aquatic genomes hinders the generalizability of these approaches.

### 3.2. AI for Habitat and Ecological Risk Assessment

Beyond species-level identification, aquatic biodiversity research requires the assessment of habitat quality, the prediction of ecological changes, and the evaluation of environmental risks. The biological research questions in this domain focus on whether machine learning can reliably model habitat suitability, forecast ecosystem changes, and assess risks posed by pollution, invasive species, and land-use alterations [56,57,58,59,60,61,62,63]. Computationally, these challenges are formalized primarily as regression and classification problems, often requiring spatial prediction and risk categorization.

Machine learning models provide flexible, data-driven alternatives to traditional correlational ecological approaches by capturing nonlinear interactions among multiple environmental variables.

#### 3.2.1. Predictive Habitat Modeling

Predictive habitat modeling addresses the biological question of whether suitable aquatic habitats for various species can be reliably predicted based on environmental parameters. From a computational standpoint, this involves supervised regression and classification tasks, where models estimate habitat suitability scores or assign habitat types using predictor variables, such as water temperature, pH, turbidity, or dissolved oxygen. A wide range of machine learning algorithms has been employed in this context, including Random Forests, Gradient Boosting Machines (e.g., XGBoost 3.0.0, LightGBM 4.6.0), and Deep Neural Networks [64,65]. These models are well-suited for capturing nonlinear and complex interactions among environmental variables, often outperforming traditional ecological approaches [66,67,68,69,70,71,72,73,74,75,76,77,78,79,80,81,82,83,84,85,86,87,88,89,90,91,92]. Random Forests are especially robust in the presence of noisy or high-dimensional data, while Gradient Boosting methods excel in structured and sparse datasets. Although Deep Neural Networks offer greater modeling flexibility, they often require large datasets and pose challenges in interpretability.

Limitations in predictive habitat modeling frequently arise from inconsistent spatial and temporal data coverage, which can lead to biased predictions. Additionally, complex models may overfit regional data and generalize poorly to new environmental conditions, while explaining their internal decision-making remains difficult for ecological validation.

#### 3.2.2. Machine Learning in Ecological Risk Assessment

Ecological risk assessment supported by AI investigates the biological question of whether vulnerable areas or ecosystems can be identified and classified based on exposure to stressors, such as pollutants or invasive species. This task is typically formalized as classification or probabilistic modeling, where risk levels are predicted using a combination of environmental and biological indicators. Ensemble learning methods, notably Extreme Gradient Boosting (XGBoost) and Bagging algorithms, have proven effective in this domain [84,85]. These models integrate heterogeneous data sources—including pollutant concentrations, land use patterns, and species sensitivity indices—to estimate the likelihood of ecological degradation [86,87,88,89,90]. By combining predictions from multiple weak learners, ensemble methods increase robustness, improve generalization, and reduce variance in high-uncertainty environments.

However, ecological risk models often face challenges, such as imbalanced datasets (few high-risk events compared to many low-risk ones), missing or noisy input data, and multicollinearity among predictors, which can obscure causal relationships crucial for designing mitigation strategies [91,92].

#### 3.2.3. AI-Based Water Quality Monitoring

AI-based water quality monitoring addresses the biological question of whether critical ecosystem parameters—such as nutrient levels or algal blooms—can be reliably predicted or monitored in real time using sensor data. Computationally, this challenge is modeled as a combination of time-series forecasting and anomaly detection, where algorithms predict future trends or flag abnormal fluctuations in water quality indicators. Deep learning models such as Long Short-Term Memory (LSTM) networks and Autoencoders have become increasingly prominent for these tasks [93,94,95,96,97,98,99,100,101,102,103,104,105,106,107]. LSTMs are particularly effective in handling temporal dependencies in sequential sensor readings, enabling accurate forecasting of parameters like turbidity, temperature, and dissolved oxygen [98,99,100,101,102,103]. Autoencoders, on the other hand, are often used for detecting anomalies that may signal pollution events or system failures.

Despite their potential, the success of these models depends on the availability of dense and high-quality sensor networks, which remain sparse in many freshwater ecosystems. Moreover, model performance can be sensitive to sensor drift, missing data, and outliers, while deep learning models’ limited interpretability often hinders their acceptance in environmental decision-making contexts [104,105,106,107,108,109,110,111].

### 3.3. AI in Remote Sensing for Freshwater Biodiversity Monitoring

Remote sensing technologies, particularly satellites and unmanned aerial vehicles (UAVs), have transformed the monitoring of freshwater biodiversity by providing large-scale, high-frequency environmental data. The central biological questions involve whether remote sensing can reliably detect ecological patterns, identify habitat changes, and monitor environmental disturbances [112,113,114,115,116,117,118,119,120,121,122,123,124,125]. Computationally, these challenges are typically formalized as image classification, change detection, and anomaly detection tasks.

Deep learning methods, particularly convolutional architectures, have been pivotal in automating the extraction of meaningful ecological information from remotely sensed data.

#### 3.3.1. Satellite- and Drone-Based AI Applications

This application addresses the biological question of whether remotely sensed imagery from satellites or drones can be used to classify aquatic habitats and detect ecologically relevant environmental changes. From a computational perspective, the challenge is typically framed as image classification or semantic segmentation, where models assign ecological labels to image regions or delineate habitat features. Convolutional neural networks (CNNs) and, in some cases, recurrent neural networks (RNNs), have been successfully employed to process and analyze satellite and drone-acquired data [119,120,121]. CNNs are particularly effective at extracting spatial features from visual inputs, enabling the detection of submerged vegetation, shoreline morphology, and water body extent [122,123,124]. When analyzing temporal sequences of images, RNNs are useful in capturing dynamic changes over time. Deep learning approaches enhance the granularity of habitat classification and allow for detailed ecological analysis even at the microhabitat level. However, several limitations remain. Satellite imagery can be hindered by cloud cover, radiometric inconsistency, and resolution constraints, especially in freely available datasets [124,125,126,127,128,129,130,131,132].

Although drones provide higher resolution, they require manual deployment, limited flight range, and complex post-processing. Moreover, deep models used in these applications require substantial amounts of labeled data and can overfit when training datasets are small.

#### 3.3.2. Pollution Detection via Remote Sensing and AI

Pollution detection through remote sensing and AI addresses the biological question of whether aquatic pollution events and degradation processes can be detected and monitored effectively using image-based data. The computational task involves anomaly detection and regression modeling, where the goal is to identify pollution signatures—such as sediment plumes, eutrophication, or oil spills—and quantify their spatial extent or severity. Supervised machine learning classifiers, including Support Vector Machines (SVMs) and Random Forests, along with CNN-based anomaly detection methods, are commonly applied to hyperspectral and multispectral satellite imagery [130,131,132]. These approaches allow for the extraction of spectral patterns associated with pollutants and enable monitoring across large spatial scales. Time-series analyses can further aid in identifying seasonal trends and detecting sudden disturbances [133,134,135,136,137,138,139,140,141,142,143,144,145,146,147].

However, the success of these methods depends heavily on access to high-resolution imagery, reliable ground-truth calibration, and the spectral distinctiveness of pollutants. In highly turbid or complex aquatic environments, misclassification risks increase, and performance may degrade without proper preprocessing or regional model adaptation.

#### 3.3.3. Land–Water Interface Analysis

The biological question addressed in this context is whether artificial intelligence methods can reliably detect and analyze changes occurring at the land–water interface, which are critical for understanding aquatic biodiversity dynamics. Computationally, this problem is formalized as change detection and segmentation, where models identify shifts in shoreline position, wetland coverage, or riparian zone configuration. Deep learning architectures, particularly U-Net and other fully convolutional networks (FCNs), have shown high effectiveness in extracting morphological features from satellite imagery and elevation data [148,149,150,151]. These models support the delineation of fine-scale aquatic-terrestrial boundaries and allow for monitoring ecosystem processes like erosion, wetland degradation, and habitat fragmentation [150,151,152,153,154,155].

Despite their utility, land–water boundary detection is challenged by seasonal hydrological fluctuations, vegetation cover changes, and atmospheric effects on imagery. Moreover, models trained in specific geographic regions may struggle to generalize elsewhere without transfer learning strategies or robust domain adaptation techniques.

### 3.4. AI in Conservation and Management Strategies

The integration of AI technologies into conservation and management efforts offers a paradigm shift toward more adaptive, data-driven decision-making in aquatic biodiversity research. The biological research questions focus on whether AI can optimize conservation interventions, enhance planning strategies, and broaden public engagement through citizen science initiatives [154,155,156]. Computationally, these tasks are typically formalized as optimization, scenario modeling, spatial prioritization, and classification problems.

By combining ecological, climatic, and socio-economic data, AI-powered systems can dynamically respond to environmental changes and policy needs.

#### 3.4.1. AI-Powered Decision Support Tools

AI-powered decision support tools address the biological question of whether artificial intelligence can assist in forecasting species responses and evaluating alternative conservation strategies. These tasks are typically formalized as predictive modeling, optimization, and decision analysis, allowing researchers to simulate ecological outcomes under different intervention scenarios. Machine learning models, such as ensemble predictors (Random Forests, Gradient Boosting Machines) and simulation-based optimizers, are frequently integrated into conservation decision-making pipelines [178,179]. These tools synthesize multiple data sources—including species distributions, climate projections, land use, and socio-economic indicators—to support adaptive conservation planning [180,181,182]. Ensemble methods are especially robust in handling heterogeneous data, while optimization algorithms like genetic algorithms or reinforcement learning frameworks can identify the most effective management strategies under uncertainty.

Nonetheless, the effectiveness of these tools depends on the quality and completeness of input data. Inadequate ecological or socio-economic datasets can introduce bias, while the complexity and opacity of some AI models may limit stakeholder engagement and interdisciplinary collaboration [183,184,185].

#### 3.4.2. AI-Assisted Conservation Planning

AI-assisted conservation planning tackles the biological question of whether artificial intelligence can optimize the design and spatial prioritization of protected areas to maximize biodiversity conservation. Computationally, this involves spatial optimization and network analysis, where algorithms are used to select areas that best meet ecological objectives under given constraints. Tools like MARXAN and Zonation, originally built on heuristic approaches, have been enhanced through the integration of machine learning and predictive habitat modeling techniques [186,187,188,189]. These AI-driven systems can simultaneously evaluate multiple objectives—such as species richness, habitat connectivity, and climate resilience—enabling more nuanced reserve design. Machine learning algorithms are also used to assess the performance of existing conservation areas and guide the expansion of protected networks.

However, planning outcomes remain sensitive to subjective inputs, such as target weights, conservation goals, and cost assumptions. Furthermore, computational complexity can be a barrier when processing large-scale spatial datasets, requiring specialized infrastructure and optimization expertise [190,191,192].

#### 3.4.3. Citizen Science and AI Integration

The integration of AI with citizen science initiatives addresses the biological question of whether non-experts can meaningfully contribute to aquatic biodiversity monitoring through AI-supported tools. This challenge is formalized as image classification and species recognition, where AI models embedded in mobile applications assist users in identifying and reporting aquatic organisms. Lightweight convolutional neural networks (CNNs) are frequently used in such platforms to enable real-time species identification with minimal computational demand [193,194,195].

These models are often pre-trained on extensive biodiversity image datasets and then fine-tuned for specific regional use cases. When deployed on smartphones, they allow citizen scientists to collect valuable ecological data across large geographic areas, particularly in regions where professional monitoring is limited. Crowdsourced observations, once validated, can significantly enhance biodiversity databases and improve spatial coverage. However, data quality remains a concern. Variability in camera quality, user expertise, and sampling effort introduces noise and biases, which may reduce classification accuracy and ecological representativeness.

### 3.5. Certainty of Evidence

The strength of evidence supporting AI applications in aquatic biodiversity research varies substantially across studies and application areas. To evaluate the certainty of findings, we considered several technical and methodological criteria, including the validation strategy, data diversity, model generalizability, and reporting transparency [193,194,195,196,197,198].

High confidence was attributed to studies that employed externally validated models, reported standard performance metrics (e.g., accuracy, precision, recall, and F1-score), and used diverse, multi-source datasets [199]. These works demonstrated robust model generalization across spatial, temporal, and taxonomic scales, particularly in domains such as CNN-based species image classification and LSTM-based water quality forecasting.

Moderate confidence characterized studies relying on internal validation only (e.g., k-fold cross-validation without external test sets), limited geographic scope, or imbalanced datasets that may impair model generalizability [200]. Examples include regional habitat modeling or acoustic classification efforts using restricted sample collections, where overfitting remains a concern despite promising reported accuracies.

Low confidence was associated with studies that lacked methodological transparency, did not report benchmarking against alternative methods, or minimally validated model outputs [201,202]. In several cases, the absence of cross-study comparisons, missing data handling, or unbalanced label distributions significantly reduced the reliability of conclusions, particularly in early-stage applications of deep learning to genomic sequence classification or unsupervised remote sensing analyses.

To strengthen the certainty of future findings, it is essential to adopt standardized evaluation protocols, including:

(a) Transparent reporting of model hyperparameters and training procedures,
(b) Consistent use of external and multi-environmental validation,
(c) Open-source publication of datasets and models where feasible,
(d) Adoption of cross-domain benchmarks to enable systematic comparisons.

Advancing the field will also require increased interdisciplinary collaboration between domain experts in aquatic ecology and technical experts in AI and ML, ensuring that computational advances are firmly grounded in ecological relevance.

### 3.6. Classification and Evaluation of AI Methods

To move beyond a simple enumeration of algorithms, we developed a structured classification framework for evaluating AI methodologies in aquatic biodiversity research. Each method is assessed based on the type of machine learning task it addresses, the data modality it operates on (e.g., image, acoustic, genomic, and structured data), and its strengths and limitations within the ecological application context.

The reviewed studies employed a wide spectrum of AI techniques (Table 3), ranging from traditional machine learning models to modern deep learning and transformer architectures.

#### Detailed Evaluation Insights

A. Traditional ML models (RFs, SVMs) remain prevalent for structured ecological datasets where feature engineering can encapsulate domain knowledge. They offer transparent decision boundaries but often struggle with complex, high-dimensional, or noisy inputs without preprocessing [57,58].
B. Convolutional neural networks (CNNs) dominate tasks involving raw visual input due to their hierarchical feature extraction capabilities. However, they require large, annotated datasets and are susceptible to adversarial noise or domain shifts [28,29,30].
C. Recurrent neural networks (RNNs) are well-suited for bioacoustics and time-dependent environmental monitoring but present optimization challenges, especially for long sequences, where vanishing gradients and temporal noise may impair learning [38,39,40].
D. Transformer models are emerging as promising alternatives for sequence-based ecological data (e.g., DNA, multi-sensor fusion), offering improved context modeling and parallelization advantages. Nevertheless, their application is currently limited by computational demands and the scarcity of domain-specific pre-training corpora [53,54].
E. Hybrid and explainable AI (XAI) approaches represent a critical future direction, aiming to bridge the gap between predictive accuracy and ecological interpretability. Examples include post hoc explanations using SHAP (Shapley Additive Explanations) or integrating feature attribution into CNN outputs.

### 3.7. Case Studies: Real-World Applications of AI in Aquatic Biodiversity Monitoring

Several real-world implementations demonstrate the practical potential of AI methods in aquatic biodiversity research. Each case study is analyzed based on the addressed biological question, the formalized computational problem, the applied AI technique, the achieved outcomes, and the identified limitations.

#### 3.7.1. Case Study 1: Real-Time Catch Estimation in Demersal Trawl Fisheries

This case study addresses the biological question of whether aquatic species can be detected and counted in real time during trawling operations to optimize gear deployment and reduce bycatch. The computational challenge involves object detection and tracking, specifically identifying and counting individual specimens in live underwater video streams. Avşar et al. (2023) [203] implemented a YOLOv4-based system integrated with the SORT (Simple Online and Realtime Tracking) algorithm to detect and track *Nephrops norvegicus* (Norway lobster) in trawl footage [203].

YOLOv4 was selected for its ability to perform high-speed object detection suitable for real-time applications, while SORT supported continuous tracking and counting under dynamic conditions. The system achieved a mean average precision (mAP) of 97.8% and 80.7% accuracy in live species counts.

However, its performance was sensitive to underwater turbidity, camera placement, and lighting, highlighting the challenges associated with deploying vision-based systems in variable environmental conditions.

#### 3.7.2. Case Study 2: Lightweight Fish Species Identification on Embedded Systems

This case study explores the biological question of whether fish species can be accurately identified on low-power, portable devices for use in citizen science and decentralized biodiversity monitoring. The computational task involves image classification, specifically recognizing fish species from images captured on resource-constrained embedded hardware.

Chan et al. (2022) [204] developed a lightweight YOLOv4-tiny model deployed on a Raspberry Pi 4 to identify aquarium fish species such as guppies and dwarf gouramis [204]. YOLOv4-tiny was chosen for its compact architecture, making it suitable for edge devices with limited computational capabilities. The system achieved over 90% classification accuracy and high mAP scores across tested species classes, demonstrating its feasibility for real-world, low-cost monitoring.

Nonetheless, its performance depended on well-lit and moderately controlled environments. Generalizing the system to wild aquatic ecosystems would require further retraining and dataset expansion to handle environmental variability.

#### 3.7.3. Case Study 3: Biodiversity Text Mining with Domain-Specific Language Models

This case study examines the biological question of whether structured ecological knowledge—such as species–habitat relationships—can be automatically extracted from the unstructured biodiversity literature. The corresponding computational task includes Named Entity Recognition (NER) and Relation Extraction (RE), aimed at identifying species, habitats, and ecological relationships from scientific text.

Abdelmageed et al. (2023) [205] developed BiodivBERT, a domain-specific transformer model pre-trained on the biodiversity literature, which significantly outperformed general-purpose models like BERT and BioBERT in ecological text analysis tasks [205]. By fine-tuning a transformer on biodiversity-specific corpora, the model achieved high precision in extracting ecological entities and relations and enabled the construction of biodiversity knowledge graphs.

However, broader adoption is hindered by the high computational costs associated with training such models and the limited availability of high-quality, annotated corpora for aquatic taxa.

Artificial intelligence has introduced powerful tools for advancing aquatic biodiversity research, enabling the automation, acceleration, and refinement of ecological monitoring and conservation practices. By formalizing biological questions into computational tasks, such as classification, regression, and optimization, researchers can leverage a diverse array of AI methodologies adapted to specific data modalities and ecological contexts. Although substantial progress has been made across species identification, habitat assessment, remote sensing, and conservation planning, challenges remain related to data availability, model generalizability, and interpretability. Hybrid models integrating deep learning with explainable AI techniques, as well as domain-specific adaptations of transformer architectures, offer promising future directions. Continued interdisciplinary collaboration and methodological transparency will be critical for ensuring that AI-driven insights contribute effectively to the preservation of aquatic biodiversity in an increasingly complex environmental landscape.

## 4. Challenges and Limitations

Despite the significant advancements in artificial intelligence for aquatic biodiversity research, several challenges and limitations hinder its full potential. AI applications in biodiversity monitoring, species identification, and conservation face obstacles related to data quality, model transferability, computational constraints, methodological inconsistencies, and ethical concerns. Addressing these issues is crucial to ensuring that AI-based tools provide reliable and actionable insights for biodiversity conservation.

### 4.1. Data Quality, Availability, and Bias in AI Models

One of the fundamental challenges in AI-driven biodiversity research is the quality, availability [203], and representativeness of training data [204]. AI models, particularly deep learning systems [205,206], require large, well-labeled datasets to function effectively. However, in the field of aquatic biodiversity, data collection remains sporadic, fragmented, and often biased toward certain regions or well-studied species.

The limited access to high-quality biodiversity datasets poses a major obstacle in AI implementation. Many ecological datasets are either proprietary, inaccessible due to institutional restrictions or suffer from inconsistencies in data recording methodologies. Furthermore, the use of AI in biodiversity research often relies on publicly available databases, which may lack sufficient diversity or include outdated and inaccurate records [207].

Bias in AI models is another critical concern. If training datasets overrepresent certain species or habitats while underrepresenting others, the AI system may produce skewed predictions. For example, models trained primarily on temperate freshwater ecosystems may struggle to accurately classify species in tropical environments [208,209,210]. Additionally, data bias can lead to misclassification errors, disproportionately affecting rare and endangered species that have limited available data for training.

To mitigate these issues, it is essential to develop comprehensive, standardized, and publicly accessible biodiversity datasets. Collaboration between researchers, conservation organizations, and governmental agencies can help improve data sharing and ensure that AI models are trained on diverse and representative datasets [211].

### 4.2. Model Transferability and Generalization Issues

A persistent limitation in AI-based biodiversity research is the poor transferability of trained models across heterogeneous environmental contexts. AI systems designed for species identification [212,213], habitat assessment, or ecological monitoring often perform well within the specific dataset or region on which they were trained but generalize poorly to new ecosystems [214]. This issue, rooted in ecological variability, undermines the scalability of AI applications in biodiversity science.

One of the main causes of low generalization is the high variability in environmental conditions across geographic regions [215]. Factors such as seasonal shifts [216], light availability, water turbidity [217], or background noise can significantly alter the quality of data inputs, thereby reducing the effectiveness of image recognition and remote sensing models. Similarly, AI models trained on bioacoustic datasets from one habitat often underperform when applied to different soundscapes due to changes in species vocalizations and ambient interference [218].

Furthermore, many machine learning models are strongly dependent on domain-specific features. Algorithms learn from the data they are exposed to [219], which makes it challenging to apply them to unseen regions or species without retraining [220]. While transfer learning techniques have been proposed to improve adaptability, they require additional computational resources and annotated data, which may not always be available to conservation practitioners [221].

A growing body of research, however, demonstrates that these challenges can be addressed through specific technical adaptations. For instance, Guo (2024) [222] applied transfer learning by fine-tuning pre-trained CNNs (initially trained on ImageNet) with locally gathered fish species images from Central Africa. This significantly improved classification accuracy, despite substantial ecological differences from the original training data. Transfer learning thus offers a scalable method for regional adaptation when full model retraining is not feasible.

In addition, federated learning has emerged as a promising approach for decentralized ecological modeling. Yang (2019) [223] and Saha (2020) [224] implemented federated frameworks in bioacoustic monitoring networks, where edge devices trained on site-specific audio data transmitted model updates, rather than raw data, to a central aggregator. This strategy preserved data privacy while enabling the model to adapt to region-specific acoustic profiles.

Despite these advances, barriers to widespread adoption remain. Transfer learning and federated approaches are constrained by computational infrastructure, data availability, and the lack of standardized protocols across institutions. Nonetheless, such methods point the way toward more robust, flexible, and ethically aligned AI systems for biodiversity conservation.

To improve model generalization, future AI frameworks should integrate cross-domain learning, data augmentation, and adaptive modeling strategies [222]. Where possible, federated learning architectures should be prioritized to avoid central data dependencies and promote inclusive, multi-regional model development [225].

### 4.3. Computational and Infrastructure Constraints

The deployment of AI models for biodiversity research requires substantial computational resources, which can be a limiting factor for many research institutions, particularly those in developing regions. Deep learning models [226], such as convolutional neural networks for species identification [227] or recurrent neural networks for bioacoustic analysis [228], demand high-performance computing infrastructure, including powerful GPUs and cloud computing access.

However, many conservation organizations, especially those operating in biodiversity-rich but resource-limited areas, lack access to such infrastructure. The cost of AI model training [229], particularly for deep learning applications, can be prohibitive, restricting the adoption of AI-based biodiversity monitoring systems [230,231]. Moreover, real-time AI applications, such as automated species recognition using underwater cameras [232] or drones, require edge computing capabilities to process data on-site, which may not always be available [233].

Another challenge is the energy consumption associated with AI computations [234]. Training deep neural networks requires significant computational power, leading to concerns about the environmental impact of large-scale AI applications [235]. The paradox of using AI for conservation while contributing to carbon emissions from energy-intensive computations highlights the need for more sustainable AI frameworks [236,237].

To address these computational constraints, researchers should explore lightweight AI models that can run on lower-resource hardware [238]. Techniques such as model pruning, quantization, and knowledge distillation can help reduce computational requirements without compromising accuracy. Additionally, increased investment in decentralized computing infrastructures, such as edge AI and distributed processing, can improve accessibility for biodiversity researchers in remote areas [239,240].

### 4.4. Lack of Standardized AI Methodologies in Biodiversity Research

One of the most pressing challenges in the application of AI in biodiversity research is the lack of standardized methodologies. AI models used for species classification, habitat modeling [241,242], and conservation planning often employ different algorithms [243], data preprocessing techniques, and evaluation metrics, making it difficult to compare results across studies [244,245].

Currently, there is no universally accepted AI framework tailored for biodiversity research. Researchers frequently develop custom AI pipelines, leading to inconsistencies in data collection [246], model validation, and performance reporting. This lack of standardization limits the reproducibility of studies and hinders the broader adoption of AI-based tools in conservation efforts.

Moreover, many AI-based biodiversity studies do not adequately report model limitations, such as overfitting, data biases, or in predictions. Without transparency in AI methodologies, conservation practitioners may struggle to assess the reliability of AI-generated insights for decision-making [247].

To overcome these challenges, the research community should establish best practices for AI applications in biodiversity science. Developing standardized benchmarks, shared biodiversity datasets, and open-source AI toolkits can enhance reproducibility and facilitate cross-study comparisons. Additionally, incorporating explainable AI (XAI) techniques can improve the interpretability of AI models, ensuring that their predictions are transparent and actionable for conservation planning.

### 4.5. Ethical and Policy Challenges in AI-Driven Conservation

The integration of AI in biodiversity research raises ethical and policy-related concerns that must be carefully addressed. One major ethical challenge is data privacy, particularly when using AI to monitor biodiversity in protected areas or Indigenous lands. The collection and processing of ecological data must align with ethical guidelines that respect local communities and biodiversity conservation laws.

A growing concern is the unintended exposure of sensitive biodiversity data through AI-assisted monitoring. For example, real-time geotagged images or camera trap footage could inadvertently reveal the locations of endangered species, potentially facilitating illegal wildlife trade if accessed by malicious actors [248,249]. Some open biodiversity platforms have been criticized for lacking adequate spatial data obfuscation, increasing the risk of exploitation.

The use of bioacoustic monitoring technologies, often deployed in wetlands and river systems, further raises privacy issues when devices collect ambient human sounds alongside ecological signals. In some regions, these recordings have sparked community-level concerns about surveillance, especially where consent protocols are lacking or poorly communicated.

AI models applied to environmental DNA (eDNA) data introduce additional challenges. As such, models become more powerful in detecting rare or commercially valuable species, ethical questions emerge regarding genomic privacy, the potential for bioprospecting, and disputes over data ownership and use rights.

Policy challenges also arise due to the lack of regulatory frameworks governing AI applications in conservation. Many countries lack specific legislation addressing AI-driven ecological research, leading to legal ambiguity around data governance, accountability, and AI model deployment. Moreover, the global disparity in infrastructure and technical capacity means that low- and middle-income countries may face barriers in adopting advanced AI tools equitably.

To mitigate these risks, policymakers and researchers must co-develop transparent and inclusive AI governance frameworks. Ethical AI principles, such as the CARE Principles (Collective benefit, Authority to control, Responsibility, and Ethics), should guide data stewardship, especially in contexts involving Indigenous knowledge and community-based conservation. Additionally, responsible AI practices—such as fairness-aware learning, explainability tools, and bias mitigation—should be embedded from the model development phase onward [250,251,252].

Collaborative efforts between governments, research institutions, and conservation organizations are essential to establish internationally recognized standards that ensure responsible innovation while protecting ecological and human interests. Table 4 summarizes the main ethical and technical challenges facing AI in aquatic biodiversity and proposes solutions for each domain.

While AI has the potential to transform aquatic biodiversity research, its widespread adoption is hindered by challenges related to data availability, model generalization, computational demands, methodological inconsistencies, and ethical concerns. Addressing these limitations requires interdisciplinary collaboration, investment in sustainable AI infrastructure, and the development of standardized methodologies. By overcoming these challenges, AI can play a pivotal role in enhancing biodiversity monitoring, conservation planning, and environmental decision-making for freshwater ecosystems worldwide [251,252,253,254].

## 5. Future Perspectives and Research Directions

The integration of artificial intelligence in aquatic biodiversity research has yielded significant advancements, yet several challenges remain that must be addressed to maximize its potential. Future research should focus on developing standardized AI frameworks, improving interpretability, integrating citizen science and big data, enhancing AI’s role in policymaking, and identifying emerging AI trends that could revolutionize freshwater biodiversity monitoring. These research directions will ensure that AI-driven conservation approaches are more robust, transparent, and actionable [255,256].

### 5.1. Need for Standardized AI Frameworks in Aquatic Ecology

The absence of standardized AI frameworks in aquatic ecology poses a major obstacle to reproducibility, comparability, and large-scale implementation. Current AI applications in biodiversity research rely on a diverse range of methodologies, including different machine learning models [257], data preprocessing techniques, and evaluation metrics. This variability makes it difficult to benchmark AI models and assess their effectiveness across different ecosystems.

To address this issue, the development of a universal AI framework tailored for aquatic ecology is crucial. Such a framework should provide standardized protocols for data collection, model training, validation, and reporting. The establishment of common performance metrics, similar to those used in other AI-driven domains, would enable researchers to systematically compare models and refine methodologies.

Furthermore, standardized datasets [258] and benchmark repositories should be created to facilitate AI training and testing across multiple ecosystems. Open-access databases that integrate water quality parameters [259], species distributions [260], and environmental stressors will enhance the scalability of AI models [261]. Collaboration among ecologists, data scientists, and AI researchers is essential to ensure that such frameworks align with ecological realities while maintaining computational efficiency.

### 5.2. Enhancing AI Interpretability and Explainability in Conservation

One of the most pressing concerns in AI-driven conservation is the black-box [262] nature of many machine learning models. Deep learning models, particularly convolutional neural networks and recurrent neural networks, often provide highly accurate predictions but lack transparency regarding their decision-making processes [263]. This lack of interpretability limits their adoption in ecological studies, where researchers and policymakers need to understand how AI-generated insights are derived [264].

Future research should prioritize the integration of explainable AI (XAI) techniques in aquatic biodiversity monitoring. Methods such as feature attribution analysis, attention mechanisms, and model-agnostic interpretability tools can help to demystify AI predictions. For instance, saliency maps can be used in computer vision models to highlight which features in an image were most influential in species classification [263,264,265,266,267,268,269]. Similarly, SHAP (Shapley Additive Explanations) and LIMEs (Local Interpretable Model-Agnostic Explanations) can be applied in ecological risk assessment models to provide insight into which environmental variables contribute most to predictions [267,268].

Moreover, enhancing AI interpretability is crucial for gaining stakeholder trust in conservation initiatives [269]. By making AI outputs more transparent, conservationists and policymakers will be better equipped to implement data-driven decisions with confidence.

### 5.3. Integration of AI with Citizen Science and Big Data

Citizen science initiatives have gained momentum in biodiversity monitoring, enabling large-scale data collection by engaging non-expert volunteers in species identification, habitat assessment, and environmental monitoring. However, the integration of AI with citizen science remains an underutilized avenue for enhancing freshwater biodiversity research [270].

Future studies should explore how AI models can process and validate citizen science data in real time. AI-powered mobile applications equipped with image recognition capabilities could enable volunteers to accurately identify species and report observations with minimal error [271]. These applications could leverage federated learning approaches, where AI models continuously improve by learning from decentralized datasets contributed by citizen scientists worldwide [272].

Additionally, AI should be integrated with big data analytics platforms to enhance biodiversity monitoring at regional and global scales [273]. By merging remote sensing data, climate models [274], and citizen science observations, AI-driven systems can detect long-term ecological trends, forecast biodiversity changes [275], and provide early warning signals for habitat degradation. Such integrative approaches will make biodiversity research more inclusive, participatory, and scalable [276].

### 5.4. AI in Policymaking for Biodiversity and Environmental Protection

AI has the potential to play a transformative role in policymaking for biodiversity conservation and environmental protection. By analyzing vast amounts of ecological and socio-economic data, AI models can provide policymakers with actionable insights for designing effective conservation strategies [277]. However, the adoption of AI in policy frameworks remains limited due to regulatory challenges and a lack of interdisciplinary collaboration between AI experts and environmental policymakers [278].

Future research should focus on developing AI-driven decision support systems tailored to environmental policymaking [279]. These systems could use predictive modeling to assess the long-term impact of conservation policies, optimize resource allocation for protected areas, and evaluate trade-offs between economic development and biodiversity conservation [280,281].

AI can also enhance environmental impact assessments (EIAs) by automating the analysis of human-induced pressures on freshwater ecosystems [282]. For example, machine learning algorithms could assess the ecological consequences of dam construction, agricultural runoff, or urbanization [283], enabling policymakers to implement mitigation measures proactively [284].

To ensure ethical AI deployment in conservation policy, there is a need for international guidelines and governance frameworks. Policymakers should work collaboratively with AI researchers to establish transparent and accountable AI systems that align with biodiversity conservation goals.

### 5.5. Future AI Trends in Freshwater Biodiversity Research

As AI technology continues to evolve, several emerging trends are expected to shape the future of freshwater biodiversity research. One such trend is the increasing adoption of self-supervised and unsupervised learning techniques [285]. Unlike traditional supervised learning [286], which requires large labeled datasets, self-supervised AI models can learn meaningful patterns from unstructured biodiversity data [287], making them highly suitable for analyzing complex ecosystems with limited labeled information [288].

Another promising trend is the use of AI-generated synthetic data for biodiversity modeling. Synthetic data augmentation techniques, such as generative adversarial networks (GANs), can create realistic ecological datasets to train AI models [289], addressing the issue of data scarcity in freshwater biodiversity studies. This approach can help improve AI performance in regions where biodiversity data collection is challenging [290].

AI-driven robotics [289,290,291] and autonomous monitoring systems are also likely to revolutionize freshwater biodiversity research. Autonomous underwater drones equipped with AI-based imaging and environmental sensors [292,293] can conduct continuous biodiversity assessments in remote and inaccessible aquatic habitats [293,294,295]. These systems could operate autonomously, collecting data on species distributions, water quality parameters, and habitat changes with minimal human intervention [296].

Additionally, AI-enabled multi-modal analysis is expected to enhance biodiversity assessments by integrating diverse data sources, including satellite imagery [297], acoustic recordings, genetic data, and climate models. Multi-modal AI approaches will provide a more holistic understanding of biodiversity dynamics, allowing for more accurate predictions and conservation planning [298].

Finally, the integration of blockchain technology with AI for biodiversity data management and conservation incentives may emerge as a novel trend [299,300]. Blockchain-based conservation frameworks could ensure data integrity, promote transparent biodiversity monitoring [301,302], and facilitate the creation of incentive mechanisms for local communities engaged in conservation efforts [303] (Table 5).

The future of AI in freshwater biodiversity research is filled with opportunities and challenges. Standardizing AI frameworks, enhancing interpretability, integrating AI with citizen science and big data, embedding AI in conservation policy, and exploring emerging AI trends will be crucial for unlocking the full potential of artificial intelligence in biodiversity conservation. Interdisciplinary collaboration between AI researchers, ecologists, and policymakers will be essential to ensure that AI-driven approaches are ethical, transparent, and effective in addressing the global biodiversity crisis. By addressing these future research directions, AI can become an indispensable tool for monitoring, preserving, and restoring freshwater ecosystems worldwide.

## 6. Conclusions

The application of artificial intelligence in aquatic biodiversity research has demonstrated its transformative potential in species identification, habitat assessment, ecological risk modeling, and conservation planning. AI-driven methodologies have significantly enhanced biodiversity monitoring by automating complex processes, analyzing vast datasets, and improving the accuracy of ecological predictions. This systematic review has highlighted the growing adoption of AI technologies in freshwater biodiversity research, revealing both their advantages and limitation.

A key takeaway from this review is the increasing role of AI in species identification, particularly through deep learning models such as convolutional neural networks (CNNs) and bioacoustic analysis. AI has enabled the rapid classification of freshwater organisms, including rare and cryptic species, with unprecedented precision. Additionally, environmental DNA (eDNA)-based AI applications have opened new frontiers in non-invasive biodiversity monitoring, allowing for the detection of species presence through genetic material in water samples.

In the realm of habitat assessment and ecological risk modeling, AI has proven valuable in predicting habitat changes, assessing pollution risks, and identifying emerging environmental threats. Machine learning models have been instrumental in analyzing complex ecological data, offering insights into the effects of climate change, land-use modifications and anthropogenic activities on freshwater ecosystem. However, challenges remain in ensuring that AI-generated predictions are robust, transferable across different ecosystems, and supported by high-quality training data.

Remote sensing and AI integration have facilitated large-scale biodiversity monitoring using satellite imagery, drone-based surveys, and water pollution detection models. These advancements have enabled researchers to monitor ecosystem health at spatial and temporal resolutions previously unattainable through conventional fieldwork. Despite this progress, concerns related to the accessibility of remote sensing data, computational demands, and model accuracy persist.

One of the most promising applications of AI lies in conservation planning and decision-making, where AI-powered tools have aided in designing protected areas, optimizing resource allocation, and developing adaptive conservation strategies. AI has also facilitated the expansion of citizen science initiatives, empowering non-experts to contribute to biodiversity research through AI-driven mobile applications. However, ethical concerns regarding data privacy, algorithmic bias, and the potential misuse of AI in biodiversity exploitation must be carefully managed.

Despite its remarkable potential, AI in biodiversity research faces significant challenges, including issues related to data quality, model generalization, computational infrastructure, lack of standardization, and ethical considerations. The effectiveness of AI models is highly dependent on the availability of comprehensive and unbiased datasets, which remain limited for many freshwater ecosystems. Furthermore, the lack of standardized AI frameworks has hindered cross-study comparability and reproducibility, emphasizing the need for universally accepted methodologies in AI-driven biodiversity research.

Looking ahead, future research should focus on developing standardized AI frameworks, improving model interpretability, integrating AI with citizen science and big data platforms, and embedding AI in policymaking for biodiversity conservation. Emerging AI trends, such as self-supervised learning, synthetic data generation, autonomous AI-driven monitoring systems, and blockchain-integrated conservation strategies, hold promise for further advancing biodiversity research.

In conclusion, AI has the potential to revolutionize freshwater biodiversity conservation by providing scalable, efficient, and precise tools for ecological monitoring. However, to fully harness its benefits, interdisciplinary collaboration among AI researchers, ecologists, policymakers, and conservation practitioners is essential. Addressing current limitations and ensuring ethical AI deployment will be crucial in shaping the future of biodiversity research, ultimately contributing to more effective conservation strategies and the protection of freshwater ecosystems worldwide.

## Figures and Tables

**Table 1:** Risk levels explanations.

Risk Level	Criteria (Example)
Low risk	Publicly available dataset, clear model architecture, external validation present
Some concerns	Model details incomplete, internal validation only, unclear label derivation
High risk	Proprietary dataset, no model transparency, no validation steps reported

**Table 2:** Summary of AI applications in aquatic biodiversity research.

AI Application	Methods Used	Key Benefits
Species Identification [157]	Computer Vision (CNNs) [158,159,160], Bioacoustics [161], eDNA-based AI [162,163]	Automated classification [164], high accuracy, scalable monitoring [165,166]
Habitat and Ecological Risk Assessment [167]	Predictive Habitat Mapping, ML-based Ecological Risk Models, AI-driven Water Quality Monitoring [168,169]	Predicts ecosystem changes, identifies threats, supports mitigation [170,171]
Remote Sensing for Biodiversity Monitoring	Satellites/Drones with AI, Deep Learning for Land–Water Interface Analysis [172]	Large-scale monitoring, real-time analysis, improved data accessibility [173,174,175]
AI in Conservation and Management Strategies [176]	AI-powered Decision Support Systems, AI-driven Conservation Planning, Citizen Science Integration [177]	Optimizes conservation decisions, enhances public participation, enables proactive strategies

**Table 3:** Summary of AI classes.

AI Method Type	Example Algorithms	Data Modality	ML Task	Strengths	Limitations
Traditional ML	Random Forests (RFs), Support Vector Machines (SVMs), k-Nearest Neighbors (k-NNs)	Structured tabular data (e.g., environmental variables, genetic markers)	Classification, Regression	Interpretable; robust to overfitting; low computational cost	Requires feature engineering; sensitive to class imbalance; limited flexibility
Deep Learning (CNNs)	ResNet, YOLO, VGGNet	Images (e.g., species photos, satellite and drone imagery)	Image Classification, Object Detection	High accuracy; automatic feature extraction; scalable to large datasets	Data-hungry; requires extensive labeling; low interpretability without XAI tools
Deep Learning (RNNs)	LSTMs, GRUs	Sequential/Temporal Data (e.g., acoustic signals, water quality time series)	Time-Series Classification, Forecasting	Captures temporal dependencies; effective for sequential patterns	Prone to overfitting; training instability; high computational resource demand
Transformer Models	BERT, DNABERT, Vision Transformer (ViT)	Genomic sequences, multi-modal data	Sequence Classification, Multi-Modal Fusion	Context-aware modeling; superior scalability; transferable across domains	High computational demand; scarce applications in aquatic field studies
Hybrid and XAI Systems	CNNs + SHAP, RFs + LIMEs	Various	Classification, Interpretation	Balances predictive performance with interpretability; enhances model trust	Experimental; limited standardization; low adoption in operational projects

**Table 4:** Key challenges and solutions for AI in aquatic biodiversity research.

Challenge	Proposed Solution
Data Quality and Bias	Develop comprehensive, diverse datasets, improve data-sharing frameworks
Model Transferability	Use transfer learning and domain adaptation techniques to enhance generalization
Computational Constraints	Implement lightweight AI models, invest in cloud and edge computing
Lack of Standardization	Establish standardized benchmarks and protocols for biodiversity AI
Ethical and Policy Concerns	Develop transparent governance, apply ethical AI principles, engage stakeholders

**Table 5:** Future research directions in AI for freshwater biodiversity.

Research Direction	Expected Impact
Standardized AI Frameworks	Ensures comparability and reproducibility of studies across ecosystems
AI Interpretability and Explainability	Improves trust and usability of AI-driven insights for conservation
Integration with Citizen Science and Big Data	Expands data collection and analysis through crowdsourcing and automation
AI in Policymaking	Enhances evidence-based conservation policies and decision-making
Emerging AI Trends	Explores novel AI methodologies to advance biodiversity research

## Data Availability

All data generated and analyzed during this study are available in the Open Science Framework (OSF) repository and can be accessed at: https://osf.io/y637n (accessed on 11 March 2025).

## Abbreviations

The following abbreviations are used in this manuscript:

AI	Artificial intelligence
CNN	Convolutional neural network
DL	Deep learning
eDNA	Environmental DNA
EIA	Environmental impact assessment
GAN	Generative adversarial network
GPS	Global Positioning System
ML	Machine learning
NLP	Natural language processing
PRISMA	Preferred Reporting Items for Systematic Reviews and Meta-Analyses
QUADAS-2	Quality Assessment of Diagnostic Accuracy Studies-2
RNN	Recurrent neural network
RoB 2	Risk of Bias 2 Tool
SHAP	Shapley Additive Explanations
SVM	Support Vector Machine
UAV	Unmanned aerial vehicle
XAI	Explainable artificial intelligence
YOLO	You Only Look Once (Object Detection Algorithm)
